# Preliminary Research to Assess the Possibility of Grinding Selected Plastics Using Crushers

**DOI:** 10.3390/polym16223104

**Published:** 2024-11-05

**Authors:** Paweł Ciężkowski, Sebastian Bąk, Jacek Caban, Jarosław Seńko, Mateusz Adam Waśkowicz

**Affiliations:** 1 Faculty of Automotive and Construction Machinery Engineering, Warsaw University of Technology, 84 Narbutta, 02-524 Warsaw, Poland; pawel.ciezkowski@pw.edu.pl (P.C.); sebastian.bak@pw.edu.pl (S.B.); jaroslaw.senko@pw.edu.pl (J.S.); 2 Faculty of Mechanical Engineering, Lublin University of Technology, 36 Nadbystrzycka, 20-618 Lublin, Poland; mateusz.waskowicz1@pollub.edu.pl

**Keywords:** crushing, particle size distribution, plastic waste, polymers, recycling of plastics

## Abstract

This study aims to investigate the effect of the shredding machine used on the recyclability of plastic fractions after primary crushing. This work presents a method for producing aggregates that has yet to be used in the plastics industry. This study included crushing of polymethylmethacrylate (PMMA), polyamide (PA-6), acrylonitrile butadiene-styrene (ABS), polycarbonate (PC), polystyrene (PS), and polypropylene (PP) waste in a jaw, a hammer, and a cone crusher. An analysis of the grain composition was carried out to characterize the obtained crushing products. The influence of feed size on the grain composition of the product and, only on the jaw crusher, the influence of the material used on the parameters of the crushing process was studied. This paper proposes a method to evaluate the grain composition and a way to assess plastic shredding capabilities based on machine kinematics and mechanical properties of a given material.

## 1. Introduction

During preparation of the process line and operation of the material comminution process, it is important to take into account the relevant operating parameters of the machinery in question, the most important of which are those relating to load and operating speed, as they have a major influence on the usable capability, efficiency, and costs generated during operation. These parameters mainly include physical (load, acceptable energy consumption, and weight), technological [1,2], operational [3,4,5], and economic quantities [6]. One factor that has a major influence on the reduction in plastic production costs is the reduction in operational, investment, and production costs through material recycling [7,8,9,10]. In this way, costs can be reduced in real terms, while actively influencing environmental protection.

**Figure 1:**
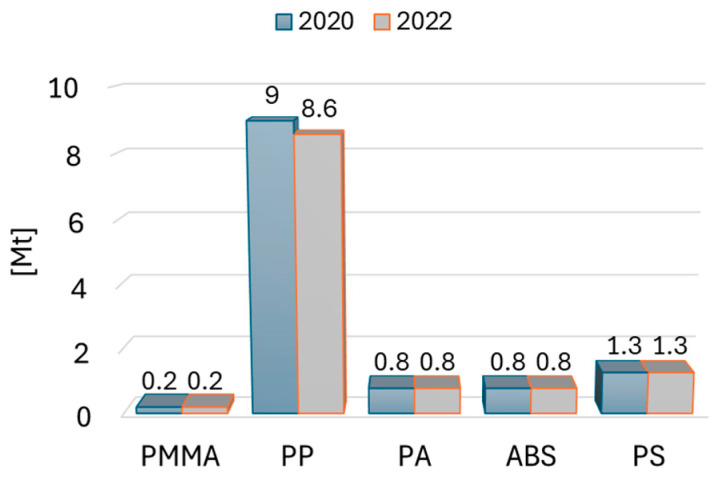
Plastics demand by resin type 2023 in Europe [15].

Recycling is now one of the main ways of managing plastics [11,12,13,14]. The huge consumption of plastic in the world and the fact that its production consumes huge quantities of non-renewable resources make it necessary to take a responsible approach to finding ways to recycle it effectively. The favorable properties of polymer plastics, including low specific weight, good mechanical properties, and ease of molding products, have resulted in their current use in all areas of industry. This has resulted in increasing demand for plastics worldwide and a rapidly increasing amount of waste, which is a growing environmental problem [15]. Currently, most countries recycle plastic, but in reality, these are very different values, and so in Europe, it is only 1–5%, while Taiwan has a recycling rate of 80%. In Europe, plastics production almost reached 58 million tons [15]. Polypropylene products are produced the most (Figure 1). The mechanical and physical properties of the materials studied in this paper are summarized in Table 1.

The increase in waste from various industries necessitates environmental protection measures. The amount of plastic in circulation is expected to increase from 236 to 417 million tons per year by 2030 [15,16,17]. Another problem is that currently, more than half of the plastics produced are disposed of without recovery [18]. The main problem is the durability of the mentioned polymers in the environment before decomposition (for example, PP decomposes in 100 years and PS in as much as 1 million years). It is likely that 12 billion tons of plastic waste will be in landfills or the environment by 2050 [15]. Instead of landfilling plastic waste, it should be recycled [19,20]. The process of mechanical recycling of polymer plastics is analyzed in the area of waste segregation and cleaning [21,22], cutting, [23,24] shredding, and secondary processing [25,26].

Plastics are increasingly replacing traditional engineering materials so that machine components can be made from them. Some polymeric plastics can be recycled and reused in the manufacturing process [27,28] and automotive sector [29,30,31]. The main way to recover polymer plastics is recycling: (a) material recycling (reprocessing of waste into a usable product) [26,32], (b) raw material recycling (recovery of raw materials used in the production of a product), and (c) energy recycling (energy recovery) [25]. Examples of recycling research work and progress in the recovery and management of plastic solid waste by various methods along with various identification/separation techniques are presented in a previous paper [33].

One type of fragmentation used in plastics processing is grinding [34,35,36,37,38]. The following machines are often used for fragmenting plastics: mills, machines with cutting blades, or cutting and jerk shredders. The selection of design features of crushing machines [39,40,41,42,43] (the geometry of the crushing elements) and technological parameters is the result of finding out and applying knowledge about the construction and operation of crushers and implementing innovations [44,45]. The way of fragmentation, the shape and working of the blades, and the kinetic energy released during the whole process influence the properties of the obtained product [46]. Thus, the quality of an aggregate is determined by both its granulometric composition and grain shape, as well as its physical and mechanical properties.

Crushing is a basic processing process used in the production of aggregates, feed and food industry, [47,48,49,50,51] and the recycling of plastics. Apart from the crushing method, the mechanical properties of the grain have a significant influence on the outcome of the process [52].

In this study, tests were conducted on three basic types of crushers (jaw crusher, cone crusher, and hammer impact crusher). The dominance of one of the destructive mechanisms (i.e., compression, abrasion, fracture, or impact) determines the energy consumption and crushing effect. For example, in jaw crushers with straight movement of the jaw, the dominant process is compression [53,54,55], and in hammer crushers, it is impact [40].

Crushing is an expensive process due to high energy consumption [56,57] and rapid wear of the working parts of the crushing equipment [58]. In order to be efficient, a certain degree of fineness suitable for the type of crushing machine should be selected in a considered manner [59]. As shown by studies on rocks, the use of unsuitable crushers results in a decrease in the quality of the obtained product [58,60].

The implementation of new crushing machines enables a better understanding of these processes [56,58,59,61,62,63]. Thanks to these implementations, it is possible to adapt the obtained variables to the form of mathematical models of crushing, [64,65,66,67,68] improve the usable systems of comminution [69,70,71,72], and increase crushing efficiency. This helps to acquire new knowledge of crushing phenomena and improve the efficiency of crushing of various materials [73,74,75]. Improper selection of the type of crushing equipment, its size, and its mode of operation for a given material can result in high energy consumption, poorer product quality, increased undesirable fractions, and faster wear of machine working components [57,59,76,77,78].

The abovementioned studies have provided relevant knowledge about rock crushing. However, there is a lack of publications discussing the applicability of crushers in plastics processing. Therefore, an interesting problem to be solved is to study the effect of the plastic comminution process on product particle size.

In this study, an attempt is made to fragment the material waste from polymer injection molding technology. This paper presents the influence of the crusher used in the crushing systems on the obtained product grain composition and degree of fineness. The process of crushing plastics was studied using a jaw crusher, a cone crusher, and a hammer crusher. The grain shapes of the obtained product using various crushing methods were discussed. The main objective of this study was to investigate how the dimensions of individual grains are formed in the product. The next task in this study was to investigate the effect of plastic grain loading on the basic parameters of the crushing (fracture) process, i.e., on the boundary forces, crushing energy, and fracture forms. For this purpose, an experimental analysis of the problem of compression of grains with coaxial punches was carried out. This study allowed for determining the displacement of crushing plates and cones favorable for the four criteria for determining the distinguishability of materials. The proposed test method is simple, fast, and efficient and can be widely used because it is reproducible in laboratory tests.

Another novelty is finding the dependence of the product grain size composition on the type of crusher used (crushing method, adopted technological line). As a result of this research, it was determined whether crushers can be used to crush plastics at the first stage of crushing. The obtained result can be used to select the optimal values of control parameters in order to improve crushing efficiency, as well as to develop automated systems for controlling plastics crushing processes in the future.

## 2. Materials and Methods

Laboratory tests were divided into three parts: In the first part, the hardness of plastics was determined. In the second part, tests were carried out using different types of crushers in order to evaluate their application in the process of plastic crushing. In the last part, detailed tests were carried out using a jaw crusher. The aim of this experiment was to determine technological parameters such as performance, crushing energy, and grain composition of the product. This paper presents the results of a comparative study of one-stage and two-stage grain crushing in hammer, cone, and jaw crushers. The quantities compared were the grain composition of the obtained product, the efficiency of the crusher, the crushing time, and the degree of fineness. The tests carried out showed that it is more favorable to crush grain in a hammer crusher. The energy consumption of the crushing process (specific energy) of PMMA and PP was also determined for a jaw crusher equipped to measure the work and crushing force [77,79]. The effect of the stamp pitch (the notch of the crushing plate) on the crushability of plastics in jaw crushers and cone crushers (which use compression as a crushing mode) was determined.

In search of a fragmentation method for plastic waste, detailed studies were carried out using materials from one of the companies involved in plastics processing and production of reflective devices for the automotive and road construction industry. The research and analysis of selected plastic crushing processes was carried out in at laboratory scale. Using available crushers (laboratory machines: hammer crusher, jaw crusher, and cone crusher), technological lines were selected, allowing us to assess the possibility of testing the machines in terms of crushing and the product obtained. The research also used a Ø300 mm LPzE-3e laboratory shaker, a WLC 30/F1/R precision scale and measuring equipment made by the Polish company MULTISERW-Morek (Marcyporęba, Poland). LabVIEW (32-bit) software was used to record machine parameter measurements.

**Figure 2:**
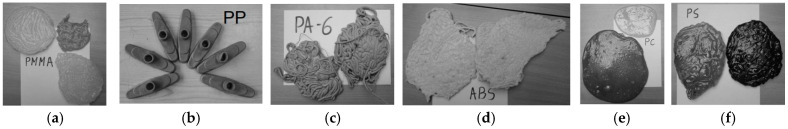
Examples of waste plastic grains: (**a**) polymethylmethacrylate PMMA—“acrylic glass”; (**b**) polypropylene PP; (**c**) polyamide PA-6; (**d**) acrylonitrile–butadiene–styrene ABS; (**e**) polycarbonate PC; (**f**) polystyrene PS.

Studies on plastics recycling processes were carried out using six types of plastic Figure 2. The hardness of the tested plastics was measured by the Shore method according to [80] with the use of a D-type hardness tester. The results obtained are presented in Table 2.

**Figure 3:**
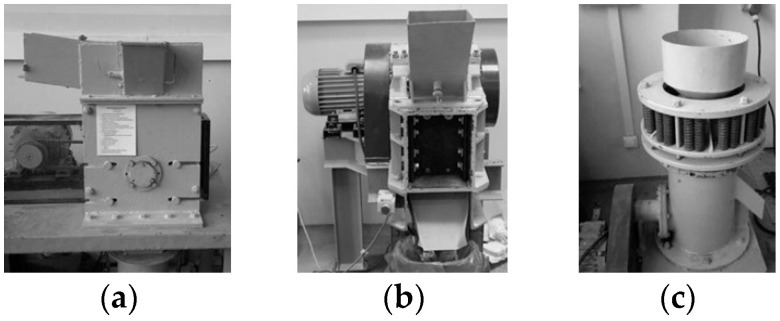
Laboratory machines: (**a**) hammer crusher, (**b**) jaw crusher, (**c**) cone crusher.

The machines used in this study are shown in Figure 3 and their operating parameters are given in Table 3.

The feed used in this study (Figure 2) was characterized by grain sizes ranging from 30 mm to 150 mm and 100% proportion of irregular grains. These are grains in which the ratio of grain length dimensions a to grain thickness c is greater than 3 (a/c > 3).

**Figure 4:**
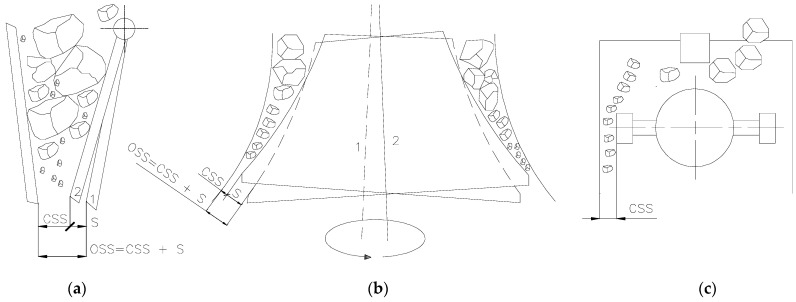
Schematic representation of crushing of plastics: (**a**) jaw crusher, (**b**) cone crusher, (**c**) hammer crusher; 1—OSS = CSS + S; 2—CSS; S—stroke.

The materials shown in Figure 2 were crushed, and the resulting product was qualitatively evaluated. Figure 4 shows a schematic comparison of crushing the material using a jaw crusher, a cone crusher, and a hammer crusher. This subject and the process of rock crushing have been dealt with, among others, by the authors of works [81,82]. Figure 4c shows how the CSS dimension was determined for hammer crusher, assumed to be the shortest distance between the hammer and the impact plate.

**Figure 5:**
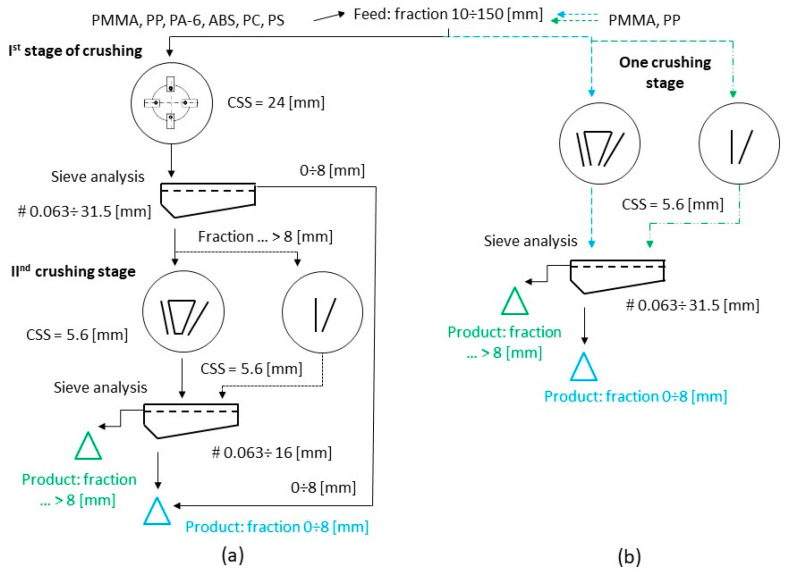
Technological scheme of plastic waste crushing and its classification: (**a**) two-stage process; (**b**) one-stage process.

Figure 5 presents the technological systems of the crushing processes, which were selected in order to analyze the suitability of the crushers for use in plastics recycling. This study was carried out for two variants of crusher operation. In the first case, the two-stage system was applied, while in the second case, the material was crushed in one stage.

In the processes where two crushers were used, in the first crushing stage, the hammer crusher (Figure 5a) operated with a minimum outlet slot (throw) CSS = 24 mm, while in the second crushing stage, jaw or cone crushers operating with equal outlet slots CSS = 5.6 mm were used to compare the results. In the hammer and jaw crusher, the feed opening was 100 mm × 200 mm. In the cone crusher, the feed size 7 was limited to D < 45 mm. After the feed was crushed in the hammer crusher, the product was screened, and grains larger than 8 mm were crushed in the jaw crusher or cone crusher.

In the second crushing variant (one-stage process), it was investigated whether the material could be crushed using only one crusher. The material shown in Figure 2a,b was selected for testing, and the crushing process was carried out using either a jaw crusher or a cone crusher (Figure 5b).

In the hammer crusher, samples were placed in a hopper over the rotor and then the material fell vertically downward onto the rotor in a uniformly accelerated motion. The wall on which the material was ejected by the rotor was perpendicular to the base.

It is well known from theoretical studies and research that the crushing efficiency of any material depends on the feed grain size and the ratio of the average diameter of the feed to the average diameter of the crushed product (degree of fineness). Tests carried out on various types of crushers and on different materials allow for experimentally determining, among others, dependencies between the effectiveness of the crushing process, the type of feed, and the degree of fineness. Establishing such relationships in conjunction with theoretical considerations allows for sensible design of machines, equipping them with an electric motor with the appropriate power and optimization of the machine mass.

Tests were carried out for three types of machines: PMMA, PP, PA-6, ABS, PC and PS were crushed, the measurements consisted of determining the crusher performance and grain composition of the product. Crushers operating at the first and the second crushing stage, when the feed fed to the crusher was pre-crushed, were studied.

## 3. Results

On the basis of the obtained results, it was revealed that the analyzed parameters of the achieved products described by grain composition, grain shape, and degree of fineness change with the change of the crushing technology, type of crusher used, and the feed used.

### 3.1. Two-Stage Crushing Process

Each stage is characterized by its inherent degree of fineness, [83] which we define as the quotient of the representative grain size of the feed *D_i_* and the product *d_i_*:

(1) $$n_{i}=\frac{D_{i}}{d_{i}},$$

where *i* = 1, 2, …—crushing stage.

Degree of fineness is equal to

*n* = *n*_1_∙*n*_2_∙*n_k_*…, (2)

where *k* denotes the number of crushers used (number of crushing stages).

To determine the degree of fineness, 80-percent grain was used as the representative grain, i.e., *D*_80_ and *d*_80_.

One of several parameters used to evaluate the crushing process is the degree of fineness, which can be determined in many ways [44,81,84]. In this paper, an 80-percent degree of fineness was determined, which is defined as the ratio of feed grain *D*_80_ to product grain *d*_80_ read from grain composition graphs (Equation (1)).

(3) $$n_{80}=\frac{D_{80}}{d_{80}},$$

On the basis of research conducted on the comminution of plastics in a two-stage process, the greatest degree of fineness was obtained for PMMA plastic and a set consisting of a hammer crusher and a cone crusher, and it amounted to *n*_2_ = 4.15 ∙ 3.99 = 16.56. The research first compared two processes in which a hammer crusher was used for the first stage of crushing, while a jaw crusher and a cone crusher were used for the second stage (Figure 5a).

At the first crushing stage, samples were fed to the hammer crusher with D80 for each material: PMMA—69.3 mm; PP—64.2 mm; PA-6—62.3 mm; ABS—70.6 mm; PC—67.9 mm; PS—68.1 mm. The influence of the type of plastic on the values of the product *d*_80_ and the degree of fineness *n*_80_ obtained during crushing with the hammer crusher is shown in Table 4. At the second crushing stage, the jaw crusher and cone crusher were fed samples for which *D*_80_ was PMMA—22.54 mm; PP—27.63 mm; PA-6—26.02 mm; ABS—43.91 mm; PC—40.86 mm; PS—24.65 mm. Each series consisted of eight measurements. The results of the influence of the machine used on the values of the obtained product *d*_80_ and the degree of fineness *n*_80_ are shown in Table 5.

It was observed that the type of plastic used influences the degree of fineness. The highest degree of fineness was obtained for PMMA, while two materials, PC and ABS, obtained the lowest values of this parameter.

The experiments show that the use of cone crusher in the crushing of plastics is ineffective compared to other types of crushers used in this processes. It was observed that ABS and PC could not be crushed: the materials jammed and blocked the rotation of the cone. During the crushing of PS, it was observed that the resulting product had a higher temperature compared to the temperature of the feed entering the crusher chamber.

According to [85], polymeric materials can be distinguished as thermosets and thermoplastics. Polymers classified as thermosets refer to the irreversible polymerization and this type of polymer is cured by chemical reaction or heat and becomes infusible and insoluble material. Thermoplastics are made up of linear molecular chains, and this polymer softens upon heating and hardens when cooled [85,86,87]. The degree of fineness of the tested plastics was higher when crushing was caused by impact (hammer crusher).

### 3.2. One-Stage Crushing Process

Table 6 shows the results of product grain size *d*_80_ and degree of fineness *n_80_* obtained from the one-stage crushing process of PMMA and PP.

The 80-percent degree of fineness was the highest and also unusual for a product obtained after a single crushing stage in a cone crusher. For the PMMA and PP aggregate studied, the degree of fineness was 12.64 for PMMA and 8.89 for PP, when usually for cone crushers it does not exceed 3–5 for rock crushing [59]. The reason for this phenomenon is the elongated shape of the feed grains. Analyzing the two-stage crushing process, the highest degree of fineness of 4.15 for 80-percent grain was obtained for the hammer crusher on the first crushing stage, and on the second crushing stage, the highest degree of fineness 3.99 was achieved for PMMA on the cone crusher, while for the jaw crusher, this parameter for the same material was equal to 3.04. Manufacturers in the case of rock crushing recommend, for reasons of the best possible parameters of the crushing process, to use a degree of fineness for jaw crushers from 3 to 4 (max. 8), for cone crushers (primary crushing) from 4 to 7 (max. 15), for cone crushers (secondary crushing) from 3 to 4 (max. 8), and for hammer crushers from 10 to 30 (max. 40) [59].

### 3.3. Particle Size Distributions

**Figure 6:**
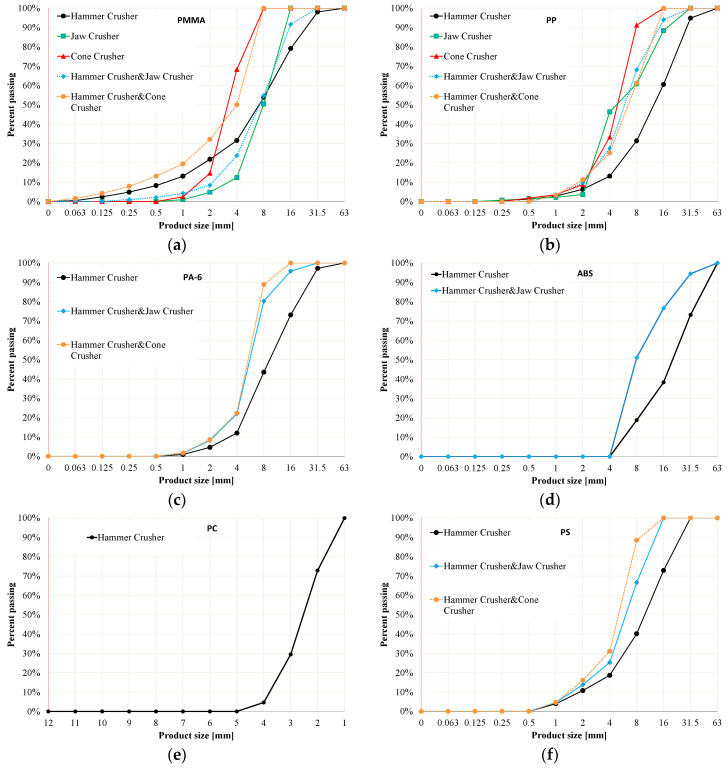
Grain composition curves of (**a**) PMMA products, (**b**) PP products, (**c**) PA-6 products, (**d**) ABS products, (**e**) PC products, and (**f**) PS products.

The graphs in Figure 6 show the results of tests on the granulometric composite on of the crushed plastics depending on the types of crushers and methods used. A set of ten sieves with square mesh sizes 0.063, 0.125, 0.25, 0.5, 1, 2, 4, 8, 16, and 31.5 mm was used.

Analyzing the achieved results, it can be observed that the number of obtained grains in a given fraction is influenced by the chemical composition of the material determining the crystallographic structure of plastic, and by the technological process adopted, which affects the shape of the produced grains [88,89] and the degree of material fineness. In the case of the hammer crusher, more than 70% of the product smaller than the outlet slot (*CSS* = 24 mm Figure 5a) was obtained for the five tested materials. The worst was ABS, for which about 50% of the product smaller than the gap was obtained.

The jaw and cone crushers failed for most of the tested plastics as first-stage crushers because they did not crush the feed. For the second crushing stage, for the jaw crusher, the following products smaller than the outlet slot (*CSS* = 5.6 mm. Figure 5a) were obtained: ABS—45%; PP—80%; PMMA—60%; PA-6—70%; PS—75%. For the cone crusher, the values were PMMA—100%; PP—50%; PC—85%; PS—70%.

### 3.4. Grain Shapes

The efficiency of the crushing process depends, among other things, on the size of the crushed feed, product quality requirements in terms of granulometric composition and grain shape, as well as on the type of machines used, their design and operating parameters [84], and the type of technological system [45,77,78,90,91].

In studies conducted on jaw and hammer crushers, the authors noted that when crushing brittle materials, the type of crusher affects the shape of the grain obtained. For example, when choosing a jaw crusher, the obtained product will be dominated by elongated grains, while when choosing a hammer crusher, cubic grains will dominate. The shape of the product obtained depends on the way the crusher operates (its type) [92,93], the amount of crushing, and the way the machine is fed [93,94].

**Figure 7:**
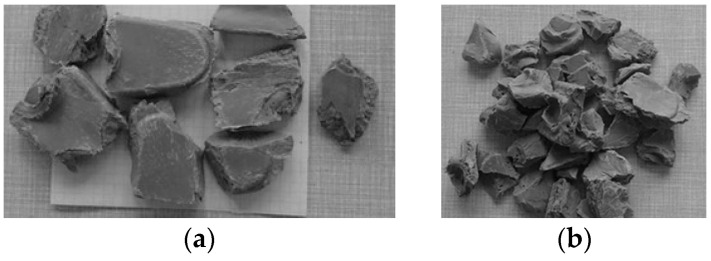
Hammer crusher, grains of PP product: (**a**) fraction … < 31.5 mm; (**b**) fraction 16–31.5 mm.

**Figure 8:**
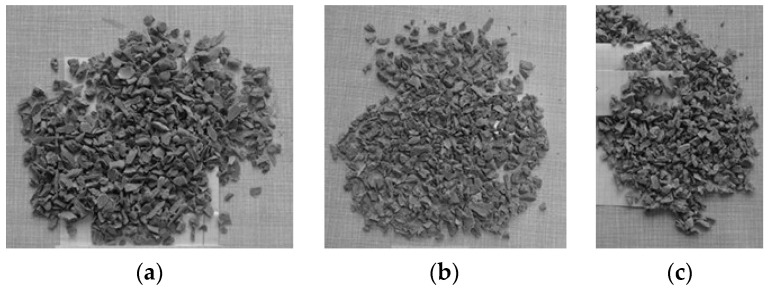
PP product, fraction 4–8 mm: (**a**) hammer crusher; (**b**) cone crusher; (**c**) jaw crusher.

Figure 7 shows the grain shape of the product obtained during PP crushing in the hammer crusher. Figure 8 shows a comparison of the product grains with a fraction of 4–8 mm obtained during crushing with a hammer crusher at the first crushing stage with the grain shape at the second crushing stage where a jaw crusher and a cone crusher were used.

**Figure 9:**
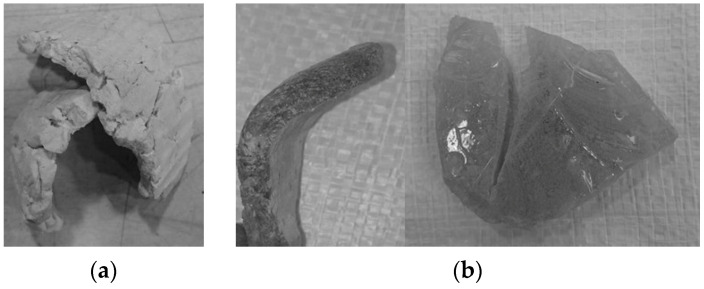
(**a**) Jaw crusher, ABS product, fraction … > 31.5 mm; (**b**) hammer crusher, grains of PC product, fraction … > 31.5 mm.

During the crushing of ABS and PC, an unusual product grain shape remaining on the 31.5 mm sieve was observed, as shown in Figure 9.

In the case of the hammer crusher, the tests confirmed for plastics that, as for rocks, a product with a cubic shape and a minimum of fine particles can be obtained.

### 3.5. Parameters Describing the Crushing Process in a Jaw Crusher

The next stage of this research was an energy assessment of the crushing process. A laboratory jaw crusher equipped with a proprietary measurement system described in previous works [77,78] was used for this study. The assessment of the amount of energy required to crush a given material is an important parameter to be taken into account when designing a technological system, and, in particular, the selection of equipment both in terms of type and size. On a laboratory scale, it is possible to experimentally estimate the energy expenditure required for crushing, and this can be achieved by measuring the power consumed by the motor, while in this paper, this is performed by measuring the force in the front toggle plate and the displacement of the jaw. The measurement system is presented in [77,78].

**Figure 10:**
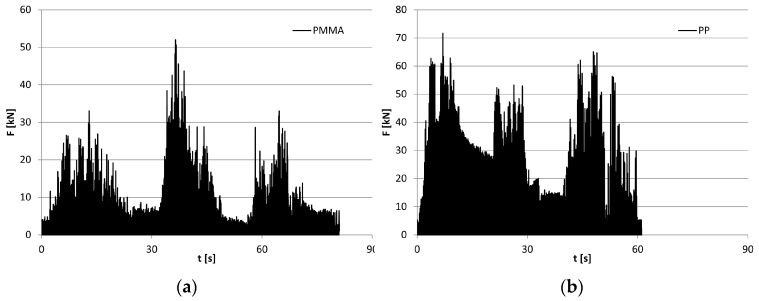
Examples of force F vs. time t plots during crushing of (**a**) PMMA; (**b**) PP.

Figure 10 shows an example of the force waveforms recorded in the toggle plate for the crushing of the PMMA and PP feed in the jaw crusher (Figure 2b).

The tests were recorded with a time step of 2 ms. The experimental results of the crushing process for two types of plastics are shown in Table 7. During the tests, the displacement of the moving jaw and the forces were recorded by measuring on the toggle plate. [77] The table gives the experimental data of the results of the average indices for specific energy Ls and technical performance Wt. The method of their determination is presented in previous works [77,78].

Analyzing the plots shown in Figure 10, the oscillation behavior of crushing forces, which is closely related to the structure of the materials, can be noticed. The more elastic-plastic the material is, the longer the crushing time, and the waveforms obtained during comminution of brittle materials are cyclic [77,78]. Disturbances observed in the plots of the crushing process adversely affect the performance of the machine and the crushing energy.

This paper presents an important issue, which is the comparison of the quantitative share of effective energy and energy returned to the system in the global energy. These results are also listed in Table 7. During crushing of plastics, there is a greater difference in the shares of the analyzed energies than in the case of crushing of rocks [77,78], where the share of energy returned to the system is observed at a level of about 13–30%.

### 3.6. Proposal for a Description of Grain Size

There are many methods by which the dimensions of grains or their surface area before and after crushing are determined [95,96,97,98]. The dimensions of a set of feed or product grains are described by a grain distribution curve. The most commonly used grain distributions are the GGS (Gates–Gaudin–Schuhmann) power distribution, the RR (Rosin–Rammler–Sperling–Bennett) distribution, the Kolmogorov distribution, and other distributions [99,100].

**Figure 11:**
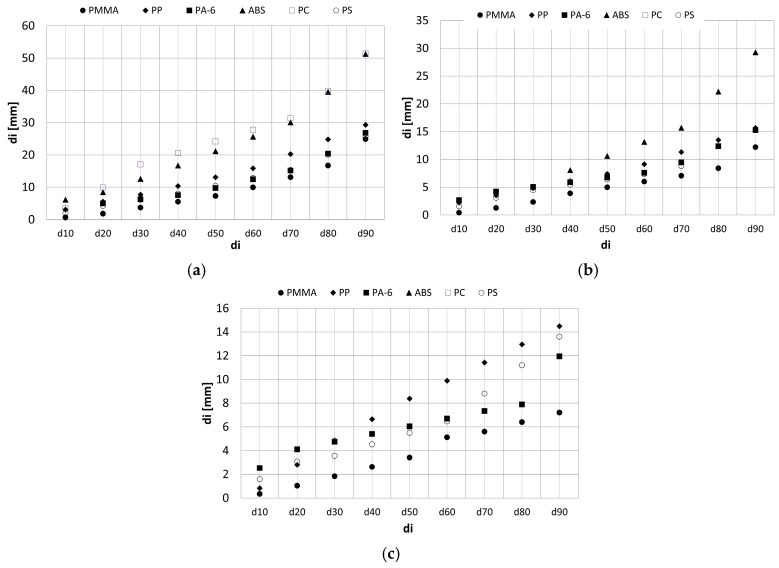
(**a**) Hammer crusher, 1st crushing stage, grain dimensions *d_i_*. (**b**) Jaw crusher, 2nd crushing stage, grain dimensions *d_i_*. (**c**) Cone crusher, 2nd crushing stage, grain dimensions *d_i_*.

When designing machines, it is important for the designer and user to know how much of a product they can obtain. In this paper, the grain size distribution is shown in Figure 11.

Grain composition is an important feature of loose materials, it has a major influence on the physicochemical properties of these materials, and these are important parameters in technological processes. So far, no single universal method for its measurement has been found. In fact, the most useful methods are these, which are based on determining the sieve diameter *dt*, effective diameter *d_e_*, substitute diameter *d_z_*, and statistical diameter *d_st_*.

Graphs are a very common tool for visualizing the analysis of product grain composition determinations. In order to show the difference in grain composition when crushing plastics with different types of crushers, a simple method was selected and proposed to compare the ratio of representative average product grain sizes. In this paper, to describe *d_i_* grain distribution, the following equations are used:

y = ax^e^, (4)

y =ax + b,(5)

where *y*—value of *d_i_* (mm); *x*—*d_i_*, in which *i* = 10, 20, …90; *a*, *b*, *e*—curve coefficients.

The values of coefficients in Equations (4) and (5) obtained for approximation of the acquired data are given in Table 8, Table 9 and Table 10. As can be seen from the results, the lowest value of the R2 coefficient was recorded for the crushed PA-6 material, regardless of the crusher used.

**Figure 12:**
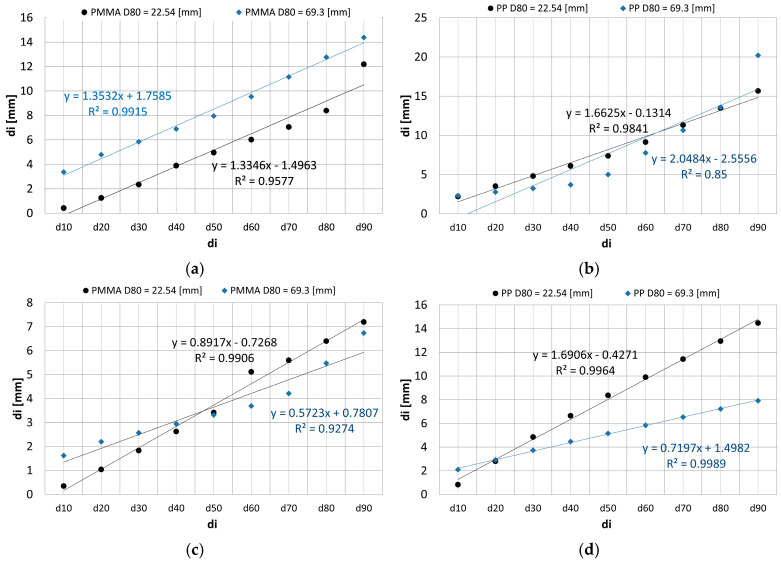
Jaw crusher, product for 1st (*D*_80_ = 69.3 mm) and 2nd stage (*D*_80_ = 22.54 mm): (**a**) PMMA; (**b**) PP. Cone crusher, product for 1st (*D*_80_ = 69.3 mm) and 2nd stage (*D*_80_ = 22.54 mm): (**c**) PMMA; (**d**) PP.

Figure 12 compares the obtained distribution of grains di in the first and second crushing stages for the jaw and cone crusher.

The grain size of the product of crushing both materials is affected by the dimension of the feed.

### 3.7. Remarks on the Fracture Modes of a Certain Plastic Under Compression

**Figure 13:**
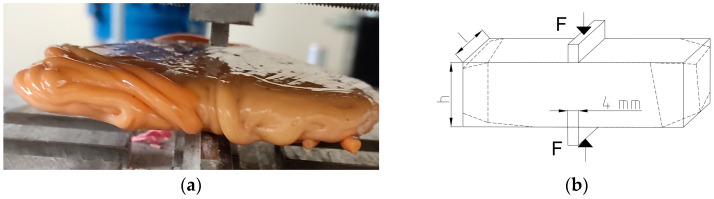
Crushing of plastic samples between coaxial punches: (**a**) PS material; (**b**) loading scheme of specimens used in the tests. l—specimen length; h—specimen height.

The intersection of a block of plastic medium by two coaxial stamps under a plane strain condition has been covered extensively in the literature. The issue has been presented by, e.g., [78,101] and many other authors. In order to evaluate the possibility of crushing plastics in static action crushers, an experiment was performed. Jaw crushers work by crushing the material between rigid plates. The wedge-shaped or narrow stamps on the plates facilitate crushing. An important scientific task is the analysis of crushing processes. In the simplest case, it consists of compressing blocks with stamps. In this paper, the simulation of the crushing process was carried out using the example of block compression with flat punches. This research was carried out on a testing machine in which specialized equipment was used to study elementary processes of crushing [62]. The subject of this research was the analysis of the process of loading non-rectangular samples of six or five plastic specimens with two coaxial flat punches with a stamp width equal to 4 mm (Figure 13). The loading process was quasi-static. In the tests, the displacements of stamps causing cutting of specimens of different heights were determined and compared to the displacement of the laboratory crusher plate. With the plots of force acting on the stamp and displacement of it for different sample heights, the ability to break the tested plastics was evaluated.

**Figure 14:**
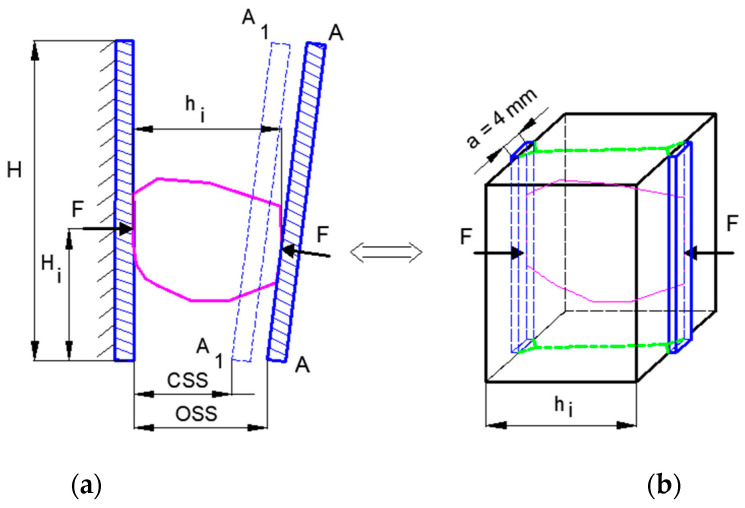
Crushing process in jaw crusher: (**a**) feed crushing scheme; (**b**) simplified sample crushing scheme. H—height of crusher working space [78].

Figure 14 shows a cross-section through the crushing chamber and the two reversible positions of the movable jaw, AA and A1A1. Figure 5 shows the maximum strain of the feed grain that occurs between the crushing plates of the jaw crusher, depending on the height of the feed grain, and the displacement of the moving plate, which depends on the height of the working chamber of the crusher for the outlet slot CSS = 5.6 mm and 18.6 mm. It can be concluded that for most of the studied materials, the crusher jaw displacement is not greater than the value of the displacement needed to crush (cut) the feed.

**Figure 15:**
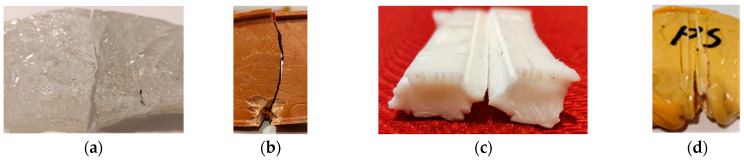
Fracture mechanisms: (**a**) PMMA; (**b**) PP; (**c**) PC; (**d**) PS.

The experimental analysis of the elementary crushing process shows that different failure mechanisms occur depending on the material used. In the case of PMMA, the specimens split in half (Figure 15a); in the case of PP specimens, a compressed zone appeared under the punch followed by specimen cracking (Figure 15b); and in the case of PS and PC, the punches compact and press the material underneath, which moves together with the punches (Figure 15c,d).

Table 11 shows the force values of crushing forces *F*, crushing energy, displacement of punches, and the value of limit loads S_r_*. When analyzing the results, the parameter S_r_* of equivalent tensile stress was introduced, analogous to the tensile stresses in the Brazilian test [102]:

(6) $$S_{r}^{*}=\frac{2F_{max}}{\pi lh},$$

where *F_max_*—limit force; *l*—specimen length; *h*—specimen height.

**Figure 16:**
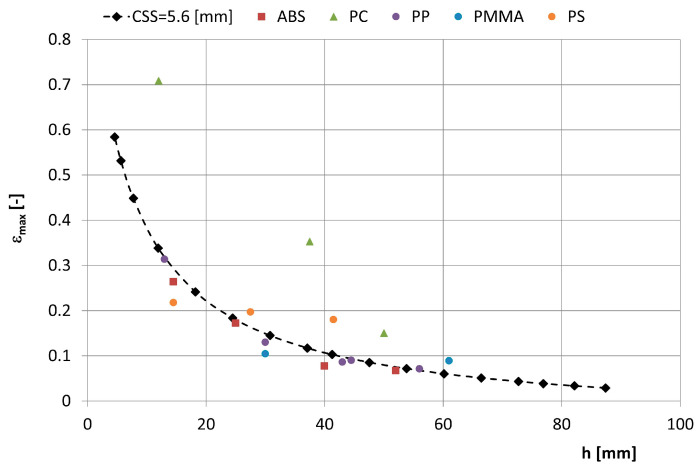
Maximum feed grain deformation that occurs between the crushing plates of the laboratory jaw crusher depending on the chamber height. h—feed grain height.

Figure 16 shows the dependence of the maximum deformation in the feed grain between the crushing plates of the laboratory jaw crusher depending on chamber height.

From the figures presented in Section 3.3, it can be seen that the hammer crusher is suitable for crushing every type of tested material. The situation is different for the jaw and cone crushers—these two machines cannot cope with all materials. During the tests, there were cases of clogging of the crusher’s outlet slot or the immobilization of the machine by residual material. Depending on demand, the material that is crushed with conventional crushers should go to the next recycling stage and be processed to the appropriate size to prepare a full-value product for reuse.

As can be seen from Figure 2, the grains take on a differentiated shape before crushing. After the crushing process, the shape of grains is uniform (Figure 7 and Figure 8), which is a result of this process. The presented results of crushing of plastics show that in the case of processing by a hammer crusher, for most materials, the percentage share of fractions smaller than 0.5 mm was very small and amounted from about 0 to 2%, which is a beneficial effect of this process.

The crusher that best prepared the feed for the mill is the hammer crusher. It produced the most grains larger than 4 mm for PC and ABS—100%; then, for PA6—88%; PP—87%; PS—82%; with the least for PMMA—68%.

PMMA is the most breakable material, while ABS and PC are the least so. To obtain more PMMA product larger than 4 mm, it is necessary to change the rotor speed of the hammer crusher, or to carry out crushing only in jaw or cone crushers.

From the comparison of the crushing products, it can be assumed that some materials have a high degree of plasticity (e.g., ABS), so they can deform very strongly without disintegrating. On the other hand, polycarbonate (PC) shows strong elastic properties; therefore, it was not crushed with the use of compressive crushers, but only strongly heated. Analyzing the results of this study, it can be seen that the hammer crusher can be used to crush plastics, and the waste recovered as aggregate can supplement the feed material for the production of plastic parts. Another advantage of using a hammer crusher in a technological system is that reducing the size of the feed grain fed to the mill will result in an increase in the efficiency of the mill.

## 4. Discussion

The methods and results presented in this paper showed that hammer crushers can be used in recycling, but operational tests should also be carried out in order to determine the influence of crushing plastics on the wear of the crusher’s components. By using impact crushers to crush plastic waste, it is possible to obtain a product characterized by good cubicity and low content of flat grains.

Based on the test results of various plastics, the following statements can be made: The displacement of the punches needed to cut the PC samples is much larger than the displacement of the crushing plate—this can be seen in Figure 16. The research shows that before selecting jaw crushers for crushing at the selection stage, it is most appropriate to carry out an analysis of the characteristics of the materials to be crushed and an analysis of the crusher jaw displacement as a function of the height of the working chamber to determine the relationship between grain height and jaw displacement causing the material to be crushed. Similar considerations apply to the cone crusher. Model studies and simulations of the crushing process by cone crushers are presented in [65,68].

Conducted research on the crushing process is incomplete and will be continued in order to obtain a more accurate assessment of the possibility of using impact crushers in plastics recycling. In future works, the problem of selecting appropriate dimensions of the rotor with hammers, flyweights, or rotating elements can be solved. An important problem is also the selection of the appropriate hammer perimeter speed for a given material (size, strength) and the desired degree of fineness. An attempt should also be made to determine the quantities that characterize the material in terms of its susceptibility to impact crushing and to theoretically explain the participation of individual elements of the crusher in the crushing process.

When analyzing the effects of plastic crushing using a hammer crusher, it was observed that despite the small number of hammers (*i* = 2), it was possible to crush most of the materials. If, in further work, it turned out that a small number of hammers could produce the same crushing effect as a larger number (e.g., four) and this conclusion was confirmed, it would be of great industrial significance, since hammers are tools that wear out very quickly.

The experimental and computational method of analyzing the process of cutting the feed with coaxial stamps is of cognitive and practical significance and has proved its usefulness in evaluating the efficiency of machine processes of plastic shredding in compressive action machines.

Further research will focus on the hammer crusher, because there are few such studies [48]. Studies can be conducted on the effect of the number of hammers and peripheral speed on degree of fineness and crushing energy; carrying out tests for more hammers (up to eight) at different speeds, from 20 to 60 m/s, and for hammers of different dimensions; carrying out tests for different inclination of the wall against which the crushed material hits; or building a model crusher so that one of the walls is transparent. This will allow observation of the processes occurring during crushing and better understanding of them, which will lead to new conclusions.

## 5. Conclusions

The results of the experiments described in this article allow for one to draw the following conclusions: as a result of the research work undertaken, a significant effect of the type of crusher and the type of plastic on the efficiency of the crushing process was found.

In the presented paper, it was also shown that the process of cutting the plastic with flat stamps can provide information on the possibility of setting the *CSS* value that will allow for a given material to be split in the crusher chamber. As the *CSS* increases, the fragmentation of the material in the working chamber will take place more easily. It is extremely important to match the recycling technology (selection of the appropriate crusher) to the type of plastic, which affects the cost-effectiveness of a given process. Crushing of plastics using crushers is, from the point of view of efficiency, a significantly less efficient method, but still effective, as proven by the obtained degree of fineness and the grain size distribution of the product. In most cases, material was obtained in the form of a collection of grains with dimensions that can be directly used in secondary processing.

The application of the proposed simple method of estimating the granulometric composition of the product showed the influence of mechanical properties of the processed material and allows for obtaining information about the percentage of individual substitute grains in the product.

## Figures and Tables

**Table 1:** Material properties [14,16].

Material	*ρ* (g/cm^3^)	*R_m_* (MPa)	*E* (GPa)	*ε* (%)	*HSh* (°Sh D)	*U* (J/mm^2^)
PMMA	1.18–1.2	60–90	2.5–4.3	2–10	83–95	18–25
PP	0.90–0.91	18–38	0.6–1.4	10–20	70–80	≈12
PA-6	1.12–1.14	38–70	1.5–3.2	4–6	80–95	50<…
ABS	1.06–1.08	30–55	1.5–2.9	2–3	80–100	50<…
PC	1.2–1.23	65–70	2–2.5	5–7	90–95	≈50
PP	1.04–1.05	40–65	3–3.6	2–4	85–90	8–18

where *ρ*—density; *R_m_*—tensile strength; *E*—Young’s modulus; *ε*—elongation at break; *HSh*—Shore hardness; *U*—impact strength.

**Table 2:** Hardness of plastics, measured by the Shore method with a D-type hardness tester (own studies).

Material	PMMA	PP	PA-6	ABS	PC	PS
Hardness (°Sh D)	76.5	74.9	67.7	78.4	78.9	77.4

**Table 3:** Basic parameters of crushers used in the tests.

	Jaw Crusher	Cone Crusher	Hammer Crusher
Inlet slot dimensions (mm)	100 × 200	≤40	100 × 200
Engine power (kW)	4	2.2	1.7
Drive shaft speed (no-load running) (rpm)	388	1410	930
Maximal feed grain size (mm)	90	30	90
CSS range (mm)	≤18.5	≤12	
Number of hammers	-	-	2
Mantle stroke (adjustable) (mm)	-	2.6–12	-
Performance for CSS = 3 mm and the feed *D_av_* = 20 mm	-	0.4 (Mg/h)	-
Mantle angle (adjustable) (°)	-	0–4.5	-

**Table 4:** First crushing stage, hammer crusher, average product grain size *d*_80_, and degree of fineness *n*_80_.

Material	*d*_80_ (mm)	*n*_80_ (-)	Time (s)	Technical Performance *W_t_* (Mg/h)	Number of Measurements in Series
PMMA	16.71	4.15	15.42	0.48	8
PP	24.76	2.59	10.67	0.59	8
PA-6	20.41	3.05	41.54	0.09	8
ABS	39.51	1.79	220.08	0.02	8
PC	39.78	1.71	111.23	0.05	8
PS	20.07	3.39	152.3	0.04	8

**Table 5:** Second crushing stage, jaw and cone crusher, average product grain size *d*_80_ and degree of fineness *n*_80_, crushing time _t_, and technical performance *W_t_*.

Material	Jaw Crusher				Cone Crusher			
	*d*_80_	*n*_80_	*t*	*W_t_*	*d*_80_	*n*_80_	*t*	*W_t_*
	(mm)	(-)	(s)	(Mg/h)	(mm)	(-)	(s)	(Mg/h)
PMMA	8.40	3.04	7.31	0.47	6.40	3.99	17.74	0.19
PP	13.48	2.05	21.6	0.21	12.95	2.13	43.20	0.10
PA-6	12.36	2.10	1.53	0.19	7.98	3.26	1.21	0.09
ABS	22.20	1.98	40.32	0.10	-	-	-	-
PC	-	-	-	-	-	-	-	-
PS	11.20	2.20	8	0.477	7.41	3.33	22.45	0.17

**Table 6:** Average product grain size *d_80_* and degree of fineness *n_80_* for one-stage process.

Material	Jaw Crusher				Cone Crusher			
	*d*_80_ (mm)	*n*_80_ (-)	*t* (s)	*W_t_* (Mg/h)	*d*_80_ (mm)	*n*_80_ (-)	*t* (s)	*W_t_* (Mg/h)
PMMA	12.77	5.43	18.5	0.4	5.48	12.64	43.54	0.17
PP	13.56	4.74	37.03	0.17	7.23	8.89	69.95	0.09

**Table 7:** Specific energy and technical performance—experiment and percentages of effective energy and energy returned to the system in global crushing energy.

Material	Specific Energy *L_s_* (kWh)	Technical Performance *W_t_* (Mg/h)	Energy Returned to the System (%)	Effective Crushing Energy (%)
PMMA	1.63	0.05	33.96	66.04
PP	0.79	0.1	52.22	47.78

**Table 8:** Hammer crusher, 1st crushing stage.

	Parameters of Equation (2)			Parameters of Equation (3)		
Material	*a*	*e*	*R* ^2^	*a*	*b*	*R* ^2^
PMMA	0.6297	1.5824	0.9680	2.7492	−4.4536	0.9180
PP	2.7511	1.01179	0.9799	3.2229	−1.6795	0.9787
PA-6	2.7395	0.8954	0.9002	2.9643	−3.3716	0.9208
ABS	4.9326	0.9566	0.9435	5.2924	−2.9627	0.9479
PC	4.0665	1.1206	0.9672	5.2851	−1.3721	0.9643
PS	1.8199	1.1293	0.9678	2.7709	−2.2261	0.9578

**Table 9:** Jaw crusher, 2nd crushing stage.

	Parameters of Equation (2)			Parameters of Equation (3)		
Material	*a*	*e*	*R* ^2^	*a*	*b*	*R* ^2^
PMMA	0.4518	1.4624	0.9746	1.3346	−1.4963	0.9577
PP	1.9528	0.8903	0.9765	1.6625	−0.1314	0.9841
PA-6	2.409	0.7280	0.8949	1.4229	0.5781	0.9280
ABS	0.8875	1.5415	0.9614	4.0950	−10.12	0.9367
PC	-	-	-	-	-	-
PS	1.649	0.8558	0.9957	1.1472	0.7097	0.9933

**Table 10:** Cone crusher, 2nd crushing stage.

	Parameters of Equation (2)			Parameters of Equation (3)		
Material	*a*	*e*	*R* ^2^	*a*	*b*	*R* ^2^
PMMA	0.3802	1.3811	0.9849	0.8917	0.7268	0.9906
PP	1.0299	1.2600	0.9843	1.6906	−0.4271	0.9964
PA-6	2.5027	0.5911	0.8513	0.9243	1.6776	0.8853
ABS	-	-	-	-	-	-
PC	-	-	-	-	-	-
PS	1.4511	0.9211	0.9347	1.4135	−0.5859	0.9433

**Table 11:** Compression with 4 mm wide coaxial stamps. Data on type of material, specimen dimensions, values of crushing energy, equivalent tensile stress, and displacement of the stamps.

Material	Specimen Height *h*	Specimen Length *l*	Limit Force *F*	Crushing Energy *L*	Equivalent Tensile Stress S_r_^*^	Displacement of the Stamps *s*	Number of Samples per Run
	(mm)	(mm)	(kN)	(J)	(MPa)	(mm)	(-)
ABS	14.5	40	8.9	8.84	9.76	3.80	8
ABS	40	36	4.69	5.81	2.07	3.10	7
ABS	52	36	4.06	7.16	1.42	3.51	7
ABS	25	36	5.31	6.26	3.67	3.70	7
PMMA	30	67	17.73	18.90	5.61	3.14	7
PMMA	61	62	12.61	25.68	2.12	5.43	8
PS	14.5	70	20.02	27.55	12.56	3.15	8
PS	27.5	45	13.67	48.72	7.03	5.42	7
PS	41.5	32	13.44	53.11	6.44	7.49	8
PC	12	60	60.03	234.87	53.07	8.5	7
PC	50	44.2	18.68	74.33	5.38	7.51	7
PC	37.5	46	33.04	297.23	12.9	13.22	9
PP	13	37	10.18	19.3	13	4.08	8
PP	43	37	10.01	18.05	4.01	3.70	7
PP	56	38	8.85	16.5	2.65	3.90	7
PP	44.5	14.5	4.65	10.63	4.63	4.0	8
PP	30	43	7.71	10,59	3.80	4.0	7

## Data Availability

The original contributions presented in this study are included in the article; further inquiries can be directed to the corresponding author.

## References

[1] Stančeková D., Sapieta M., Rudawska A., Náprstková N., Janota M. Production process intensification of a specific friction bearing by change of production technology and used material. Proceedings of the 28th International Conference on Metallurgy and Materials.

[2] Mares A., Sabadka D., Molnar V., Fedorko G. (2023). Improving competitiveness of an assembly line by simulation based productivity increase—A case study. J. Compet..

[3] Fedorko G., Molnár V., Vasi’ M., Salai R. (2021). Proposal of digital twin for testing and measuring of transport belts for pipe conveyors within the concept Industry 4.0. Measurement.

[4] Rudawska A., Madleňák R., Madleňáková L., Droździel P. (2020). Investigation of the Effect of Operational Factors on Conveyor Belt Mechanical Properties. Appl. Sci..

[5] Zhang C., Zhang Y., Dui H., Wang S., Tomovic M. (2022). Importance measure-based maintenance strategy considering maintenance costs. Eksploat. Niezawodn.-Maint. Reliab..

[6] Lukáč S., Marasová D., Mikušová N., Stopka O. (2020). Quality Management of Information Systems. Qual.-Access Success.

[7] Brzozowska J., Pizoń J., Baytikenova G., Gola A., Zakimova A., Piotrowska K. (2023). Data engineering in crisp-dm process production data—Case study. Appl. Comput. Sci..

[8] Jenis J., Hrcek S., Brumercik F., Bastovansky R. (2021). Design of Automatic Assembly Station for Industrial Vehicles Parts. LOGI-Sci. J. Transp. Logist..

[9] Marczak H. (2022). Energy Inputs on the Production of Plastic Products. J. Ecol. Eng..

[10] Sąsiadek M., Niedziela M., Woźniak W., Jachowicz T., Mikušová N. (2023). A New Effective Algorithm for Mechanical Assembly Sequence Planning. Adv. Sci. Technol. Res. J..

[11] Mikula K., Skrzypczak D., Izydorczyk G., Warchoł J., Moustakas K., Chojnacka K., Witek-Krowiak A. (2021). 3D printing filament as a second life of waste plastics—A review. Environ. Sci. Pollut. Res..

[12] Mikušová N., Stopka O., Stopkova M. (2019). Application of Multi-criteria Decision-making Methods for the Area of Recycling. TEM J..

[13] Piasecka I., Tomporowski A., Piotrowska K. (2018). Environmental analysis of post-use management of car tires. Przem. Chem..

[14] Vazquez-Velazquez A.R., Vazquez-Garcia R.A., Hernandez-Bucio G., González-González V.A., Moggio I., Vazquez-Rodriguez S. (2020). Phenylvinilbisquinolines as fluorescent markers in functionalized polypropylene films. Polym. Bull..

[15] (2024). Plastics, Plastics—The Facts 2024 The Circular Economy for Plastics A European Analysis, MARCH. https://plasticseurope.org/wp-content/uploads/2024/05/Circular_Economy_report_Digital_light_FINAL.pdf.

[16] Almeshari B., Junaedi H., Baig M., Almajid A. (2023). Development of 3D printing short carbon fiber reinforced polypropylene composite filaments. J. Mater. Res. Technol..

[17] De Fano D., Schena R., Russo A. (2022). Empowering plastic recycling: Empirical investigation on the influence of social media on consumer behavior. Resour. Conserv. Recycl..

[18] Thomson H., Illingworth K., McCoach H., Jefferson M., Morgan S. Plastic Flow 2025: Plastic Packaging Flow Data Report. https://www.wrap.ngo/resources/report/plasticflow-2025-plastic-packaging-flow-data-report.

[19] Pączkowski P., Głogowska K. (2024). Preparation and Characterization of Quartz-Reinforced Hybrid Composites Based on Unsaturated Polyester Resin from Post-Consumer PET Recyclate. Materials.

[20] Patel K.S., Shah D.B., Joshi S.J., Patel K.M. (2023). Developments in 3D printing of carbon fiber reinforced polymer containing recycled plastic waste: A review. Clean. Mater..

[21] Ragaert K., Delva L., Van Geem K. (2017). Mechanical and chemical recycling of solid plastic waste. Waste Manag..

[22] Schyns Z.O.G., Shaver M.P. (2021). Mechanical Recycling of Packaging Plastics: A Review. Macromol. Rapid Commun..

[23] Sykutera D., Bielinski M. (2019). Improving the effectiveness of the mechanical recycling processes of thermoplastics with a porous structure. Polimery.

[24] Muthiah E., Rathanasamy R., Ravichandran D., Palanichamy D., Sivaraj S. (2022). Experimental analysis on shredder for recycling thermoplastics using injection moulder. Mater. Today Proc..

[25] Khoo H.H. (2019). LCA of plastic waste recovery into recycled materials, energy and fuels in Singapore. Resour. Conserv. Recycl..

[26] Tulashie S.K., Boadu E.K., Kotoka F., Mensah D. (2020). Plastic wastes to pavement blocks: A significant alternative way to reducing plastic wastes generation and accumulation in Ghana. Constr. Build. Mater..

[27] Atzeni E., Iuliano L., Minetola P., Salmi A. (2010). Redesign and cost estimation of rapid manufactured plastic parts. Rapid Prototyp. J..

[28] Pieniak D., Samociuk W., Firlej M., Firlej E., Krzysiak Z., Przystupa K., Mańkowska-Snopczyńska A., Roszowska-Jarosz M. (2023). Comparative studies of the Knoop hardness of selected light-cured polymeric materials used for the production of spare parts working in kinematic nodes. Przem. Chem..

[29] Kurien R.A., Selvaraj D.P., Sekar M., Koshy C.P. (2020). Green composite materials for green technology in the automotive industry. IOP Conf. Ser. Mater. Sci. Eng..

[30] Vieyra H., Molina-Romero J.M., Calderón-Nájera J.D.D., Santana-Díaz A. (2022). Engineering, Recyclable, and Biodegradable Plastics in the Automotive Industry: A Review. Polymers.

[31] Zvolenský P., Kašiar Ľ., Volna P., Barta D. (2017). Simulated Computation of the Acoustic Energy Transfer through the Structure of Porous Media in Application of Passenger Carriage Body. Procedia Eng..

[32] Morici E., Dintcheva N.T. (2022). Recycling of Thermoset Materials and Thermoset-Based Composites: Challenge and Opportunity. Polymers.

[33] Singh N., Hui D., Singh R., Ahuja I.P.S., Feo L., Fraternali F. (2017). Recycling of plastic solid waste: A state of art review and future applications. Compos. Part B Eng..

[34] Avolio R., Spina F., Gentile G., Cocca M., Avella M., Carfagna C., Tealdo G., Errico M. (2019). Recycling Polyethylene-Rich Plastic Waste from Landfill Reclamation: Toward an Enhanced Landfill-Mining Approach. Polymers.

[35] Berk Z. (2018). Food Process Engineering and Technology.

[36] Köken E., Lawal A.I. (2021). Investigating the effects of feeding properties on rock breakage by jaw crusher using response surface method and gene expression programming. Adv. Powder Technol..

[37] Maris J., Bourdon S., Brossard J.-M., Cauret L., Fontaine L., Montembault V. (2018). Mechanical recycling: Compatibilization of mixed thermoplastic wastes. Polym. Degrad. Stab..

[38] Shrivastava A. (2018). Introduction to Plastics Engineering.

[39] Melicherčík J., Kuvik T., Krilek J., Čabalová I. (2021). Design of the crusher for plastic and rubber waste produced in automotive industry. FME Trans..

[40] Xue M., Li J., Xu Z. (2012). Environmental Friendly Crush-Magnetic Separation Technology for Recycling Metal-Plated Plastics from End-of-Life Vehicles. Environ. Sci. Technol..

[41] Reddy S., Raju T. (2018). Design and Development of mini plastic shredder machine. IOP Conf. Ser. Mater. Sci. Eng..

[42] Wong J.H., Gan M.J.H., Chua B.L., Gakim M., Siambun N.J. (2022). Shredder machine for plastic recycling: A review paper. IOP Conf. Ser. Mater. Sci. Eng..

[43] Rahim N.H.A., Khatib A.N.H.M. (2021). Development of PET bottle shredder reverse vending machine. Int. J. Adv. Technol. Eng. Explor..

[44] Kruszelnicka W. (2020). A New Model for Environmental Assessment of the Comminution Process in the Chain of Biomass Energy Processing. Energies.

[45] Flizikowski J., Kruszelnicka W., Macko M. (2021). The Development of Efficient Contaminated Polymer Materials Shredding in Recycling Processes. Polymers.

[46] Roh S., Jang Y., Yoo J., Seong H. (2023). Surface Modification Strategies for Biomedical Applications: Enhancing Cell–Biomaterial Interfaces and Biochip Performances. BioChip J..

[47] Soto-Gómez D., Pérez-Rodríguez P. (2022). Sustainable Agriculture through Perennial Grains: Wheat, Rice, Maize, and Other Species. A Review. Agric. Ecosyst. Environ..

[48] Polari J.J., Garcí-Aguirre D., Olmo-García L., Carrasco-Pancorbo A., Wang S.C. (2018). Impact of industrial hammer mill rotor speed on extraction efficiency and quality of extra virgin olive oil. Food Chem..

[49] Kruszelnicka W., Opielak M., Ambrose K., Pukalskas S., Tomporowski A., Walichnowska P. (2022). Energy-dependent particle size distribution models for multi-disc mill. Materials.

[50] Lomovskiy I., Bychkov A., Lomovsky O., Skripkina T. (2020). Mechanochemical and Size Reduction Machines for Biorefining. Molecules.

[51] Veillet S., Tomao V., Bornard I., Ruiz K., Chemat F. (2009). Chemical changes in virgin olive oils as a function of crushing systems: Stone mill and hammer crusher. Comptes Rendus Chim..

[52] Korman T., Bedekovic G., Kujundzic T., Kuhinek D. (2015). Impact of physical and mechanical properties of rocks on energy consumption of jaw crusher. Physicochem. Probl. Miner. Process..

[53] Johansson M., Bengtsson M., Evertsson M., Hulthén E. (2017). A fundamental model of an industrial scale jaw crusher. Miner. Eng..

[54] Legendre D., Zevenhoven R. (2014). Assessing the energy efficiency of a jaw crusher. Energy.

[55] Ozdemir K. (2021). Evaluation of blast fragmentation effects on jaw crusher throughput. Arab. J. Geosci..

[56] Eisenlauer M., Teipel U. (2021). Comminution Energy and Particulate Properties of Cutting and Hammer-Milled Beech, Oak, and Spruce Wood. Powder Technol..

[57] Rohon K., Mwema F., Dabees S., Sobola D. (2022). Advances in sustainable grinding of different types of the titanium biomaterials for medical applications: A review. Biomed. Eng. Adv..

[58] Numbi B.P., Zhang J., Xia X. (2014). Optimal energy management for a jaw crushing process in deep mines. Energy.

[59] (2021). Basics in Mineral Processing Handbook.

[60] Fladvad M., Onnela T. (2020). Influence of jaw crusher parameters on the quality of primary crushed aggregates. Miner. Eng..

[61] Vititnev A.Y., Alashkevich Y.D., Chistova N.G., Marchenko R.A. (2021). Improving the construction of grinding disk mill for producing fibrous semi-finished products. IOP Conf. Ser. Mater. Sci. Eng..

[62] Ciężkowski P. (2019). Analysis of Machine Crushing Processes.

[63] Kruszelnicka W., Kasner R., Bałdowska-Witos P., Flizikowski J., Tomporowski A. (2020). The Integrated Energy Consumption Index for Energy Biomass Grinding Technology Assessment. Energies.

[64] Cleary P.W., Delaney G.W., Sinnott M.D., Cummins S.J., Morrison R.D. (2020). Advanced comminution modelling: Part 1—Crushers. Appl. Math. Model..

[65] Atta K.T., Johansson A., Gustafsson T. (2014). Control oriented modeling of flow and size distribution in cone crushers. Miner. Eng..

[66] Machado Leite M.R. (1990). Kinetic models for the simulation of crushing circuits. Miner. Eng..

[67] Yamashita A.S., Thivierge A., Euzébio T.A.M. (2021). A review of modeling and control strategies for cone crushers in the mineral processing and quarrying industries. Miner. Eng..

[68] Atta K.T., Euzébio T., Ibarra H., Moreira V.S., Johansson A. (2019). Extension, Validation, and Simulation of a Cone Crusher Model. IFAC-Pap..

[69] Flizikowski J., Mrozinski A., Tomporowski A. (2017). Active Monitoring as Cognitive Control of Grinders Design. AIP Conf. Proc..

[70] Ostroukh A.V., Gimadetdinov M.K., Popov V.P. (2015). Selection Process Equipment for Automated Crushing Plant. Autom. Control Tech. Syst..

[71] Гимадетдинов M.K., Остроух A.B. (2014). List and Sequence of Solutions for Automated Crushing and Screening Production. Autom. Control Tech. Syst..

[72] Ostroukh A., Surkova N., Varlamov O., Chernenky V., Baldin A. (2018). Automated process control system of mobile crushing and screening plant. J. Appl. Eng. Sci..

[73] Bachér J., Rintala L., Horttanainen M. (2022). The effect of crusher type on printed circuit board assemblies’ liberation and dust generation from waste mobile phones. Miner. Eng..

[74] Ciężkowski P. (2012). Correlation of energy consumption and shape of crushing plates. AGH J. Min. Geoengin..

[75] Li J., Lu H., Guo J., Xu Z., Zhou Y. (2007). Recycle Technology for Recovering Resources and Products from Waste Printed Circuit Boards. Environ. Sci. Technol..

[76] Łączny D., Macko M., Moraczewski K., Lewandowski J. (2021). Influence of design features of a multi-edge shredder on the operational characteristics of the process of comminution corn stalks. IOP Conf. Ser. Mater. Sci. Eng..

[77] Ciężkowski P., Maciejewski J., Bąk S. (2017). Analysis of Energy Consumption of Crushing Processes—Comparison of One-Stage and Two-Stage Processes. Stud. Geotech. Mech..

[78] Ciężkowski P., Maciejewski J., Bąk S., Kwaśniewski A. (2020). Application of The New Shape Crushing Plate in Machine Crushing Processes. Stud. Geotech. Mech..

[79] Ciężkowski P., Maciejewski J., Timofiejczuk A., Łazarz B.E., Chaari F., Burdzik R. (2018). Study on Load Distribution in the Working Space of Lever Crusher. Advances in Technological Diagnostics.

[80] (2003). Plastics and Ebonite—Determination of Indentation Hardness by Means of a Durometer (Shore Hardness).

[81] Kelly E.G., Spottiswood D.J. (1982). Introduction to Mineral Processing.

[82] Köken E. (2020). Evaluation of size reduction process for rock aggregates in cone crusher. Bull. Eng. Geol. Environ..

[83] Malewski J. (2013). Licz i oszczędzaj: Projektowanie schematów technologicznych przeróbki skał. Cz. 1. Surowce Masz. Bud..

[84] Evertsson C.M. (1999). Modelling of flow in cone crushers. Miner. Eng..

[85] Grigore M. (2017). Methods of Recycling, Properties and Applications of Recycled Thermoplastic Polymers. Recycling.

[86] Ashori A. (2008). Wood–plastic composites as promising green-composites for automotive industries!. Bioresour. Technol..

[87] Ribeiro M., Fiúza A., Ferreira A., Dinis M., Meira Castro A., Meixedo J., Alvim M. (2016). Recycling Approach towards Sustainability Advance of Composite Materials’ Industry. Recycling.

[88] Choi H., Lee W., Kim D.-U., Kumar S., Ha J., Kim S., Lee J. (2009). A comparative study of particle size analysis in fine powder: The effect of a polycomponent particulate system. Korean J. Chem. Eng..

[89] Hann D., Stražišar J. (2007). Influence of Particle Size Distribution, Moisture Content, and Particle Shape on the Flow Properties of Bulk Solids. Instrum. Sci. Technol..

[90] Benabderrahmane A., Zeghloul T., Aksa W., Tilmatine A., Medles K., Dascalescu L. (2020). Shredding as simultaneous size-reduction and tribo-charging operation for improved performances of an electrostatic separation process for granular plastic wastes. Part. Sci. Technol..

[91] Głogowska K., Rozpędowski J. (2016). Examination of shredding process parameters and the properties of recyclate. Adv. Sci. Technol. Res. J..

[92] Eloranta J. (1995). Influence of Crushing Process Variables on the Product Quality of Crushed Rock.

[93] Briggs C., Evertsson C.M. (1998). Shape potential of rock. Miner. Eng..

[94] Bengtsson M. (2009). Quality-Driven Production of Aggregates in Crushing Plants. Ph.D. Thesis.

[95] Lyu F., Thomas M., Hendriks W.H., van der Poel A.F.B. (2020). Size reduction in feed technology and methods for determining, expressing and predicting particle size: A review. Anim. Feed Sci. Technol..

[96] Peszko B., Tumidajski T. (2004). Methodology of determining the dependencies between the grain composition analyses results. J. Pol. Miner. Eng. Soc..

[97] Singh M.K., Singh A. (2021). Characterization of Polymers and Fibres.

[98] Allen T. (1981). Particle Size Measurement.

[99] Colorado-Arango L., Menéndez-Aguado J.M., Osorio-Correa A. (2021). Particle Size Distribution Models for Metallurgical Coke Grinding Products. Metals.

[100] King R.P. (2001). Modelling and Simulation of Mineral Processing Systems.

[101] Hill R. (2009). The Mathematical Theory of Plasticity.

[102] García V.J., Márquez C.O., Zúñiga-Suárez A.R., Zuñiga-Torres B.C., Villalta-Granda L.J. (2017). Brazilian Test of Concrete Specimens Subjected to Different Loading Geometries: Review and New Insights. Int. J. Concr. Struct. Mater..

